# Cx26 heterozygous mutations: role in hyperacusis and vulnerability to noise

**DOI:** 10.1038/s41392-023-01525-1

**Published:** 2023-07-10

**Authors:** Karen Tawk, Mehdi Abouzari

**Affiliations:** Department of Otolaryngology-Head and Neck Surgery, University of California, Irvine, CA USA

**Keywords:** Cytogenetics, Predictive markers, Epigenetics

In a recent study published in *Science Advances*, Liu et al. demonstrated that heterozygous deletion of connexin 26 (Cx26) in mice is not harmless as they lead to hyperacusis-like hearing oversensitivity. The authors also found that children who carry a single-point heterozygous mutation in the gap junction (GJ) gene Cx26 (GJB2) are susceptible to noise, which eventually leads to hearing loss (HL).^1^

HL affects millions of people worldwide and is considered the most prevalent sensory disorder, encompassing a wide range of heterogeneous characteristics. Its incidence ranges from 1 to 2 per 1000 newborns, with approximately 60% of cases attributed to genetic causes. Non-syndromic HL (NSHL) makes up 70% of all hereditary HL cases, with the majority of cases of pre-lingual HL arising from an autosomal recessive mutation in GJB2, located on chromosome 13.^2^ Gene GJB2 encodes the GJ protein Cx26, and its mutation has been linked to cochlear development disorders.^3^ Therefore, homozygous mutations, digenic heterozygous mutations, and knockout of GJB2 can cause congenital or late-onset NSHL.^2^ Moreover, the recent study by Liu et al. challenged the long-held belief that individuals with a single recessive heterozygous GJB2 mutation are resistant to HL. On the contrary, they unexpectedly discovered that these individuals may be more susceptible to noise-induced HL and hyperacusis-like hearing oversensitivity.

**Fig. 1:**
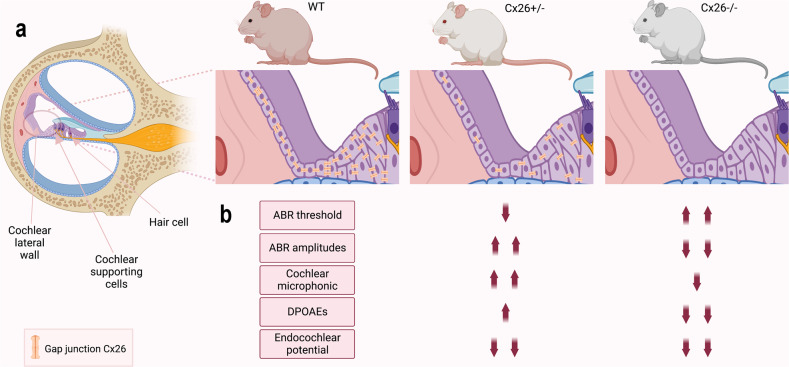
Mechanism of increase of hearing sensitivity in Pax2-Cx26^+/−^ hetero-deletion mice. **a** Diagram of gap junction (GJ) network in the cochlea of WT, Cx26^+/−^, and Cx26^−/−^ mice. **b** Cx26^+/−^ hetero-deletion in mice is associated with increased ABR threshold, larger ABR amplitudes, increased CM and DPOEs, and reduced endocochlear potential. In comparison, in Cx26^−/−^ mice, the ABR thresholds are increased and the ABR amplitudes, CM, DPOEs, and endocochlear potential are decreased. This figure was created with BioRender.com

First, Liu et al. examined whether Cx26^+/−^ hetero-deletion mice, which showed weaker Cx26 labeling in the cochlea compared to the wild-type (WT) mice, exhibited increased hearing sensitivity. They discovered that the auditory brainstem response (ABR) thresholds of Cx26^+/−^ mice were significantly lower and the amplitudes of ABR waves were significantly higher than those of WT mice and Cx26^−/−^ KO mice. These results indicated enhanced hearing sensitivity in Cx26^+/−^ mice. The authors also observed that the cochlear microphonic (CM), a receptor potential generated from the hair cells, was significantly increased in Cx26^+/−^ mice compared with WT mice, with a larger increase at a higher intensity (dB). Similarly, the distortion product otoacoustic emission (DPOAE), which represents the active cochlear amplification process, was also significantly increased in Cx26^+/−^ mice. The authors further evaluated prestin expression in the cochlea of Cx26^+/−^ mice and found that it was upregulated in Cx26^+/−^ mice in comparison to WT mice. However, the endocochlear potential was reduced by 50% in Cx26^+/−^ mice. These results suggest that HL (i.e., EP reduction) can trigger a compensatory increase in prestin expression to enhance active cochlear amplification (i.e., DPOAE). This, in turn, can amplify the vibration of the basilar membrane and result in an increase in auditory receptor currents (i.e., CM increase), ultimately leading to hyperacusis-like hearing oversensitivity in Cx26^+/−^ mice (Fig. 1). On the other hand, mice that had a heterozygous targeted deletion of the Cx26 only in the supporting cells of the cochlea but not in the lateral wall did not exhibit a reduction in EP, nor an increase in ABR or DPOAE, further supporting the previously described mechanism of hearing sensitivity and demonstrating that the hetero-deletion primarily impairs the GJ function in the cochlear lateral wall.

After noise exposure, the authors found that both WT mice and Cx26^+/−^ mice showed a significant increase in ABR thresholds, with a greater increase at the high-frequency range in Cx26^+/−^ mice. The study also found that the recovery from the noise exposure was slower and incomplete in Cx26^+/−^ mice, causing a permanent threshold shift. These findings suggest that the mutation in Cx26^+/−^ mice may have increased their susceptibility to noise and have resulted in HL. Such information is crucial for the development of effective protective strategies to prevent HL. Furthermore, the authors found that the DPOAE was significantly reduced, which was consistent with the increased ABR thresholds in noise-exposed Cx26^+/−^ mice, suggesting that HL is due to the impairment of outer hair cells’ electromotility and reduced active cochlear amplification.^3^

Moreover, the authors investigated the hearing function of 15 children who carried a single-point heterozygous mutation in the GJB2 gene and had passed the hearing screening test at birth. As observed in Cx26^+/−^ mice, they found that these carriers exhibited increased active cochlear amplification and hearing sensitivity. In addition, GJB2 heterozygote carriers had significantly increased DPOAEs and large ABRs in comparison to normal control children. Overall, these findings suggest that children who are carriers of the GJB2 mutation have increased hearing sensitivity through the same mechanism as in mice with Cx26 hetero-deletion. Genetic counseling is an important tool for individuals who carry a single heterozygous mutation in the GJB2 gene and plan to have children. Although such individuals may appear to be unaffected by HL, if both parents carry a Cx26 mutation, their child has a 25% risk of inheriting two copies of the mutated gene and being born with Cx26-related HL.^4^

In summary, Liu et al. showed that heterozygous deletion of Cx26 can cause hyperacusis-like hearing oversensitivity and increased susceptibility to noise-induced HL. The study also suggested that individuals carrying a single-point heterozygous mutation in GJB2 may have a similar mechanism of increased hearing sensitivity and vulnerability to noise. Recent advancements in the field of hearing research have shown promising results using gene therapy in the treatment of genetic hearing disorders. Moreover, the discovery of LDL receptor-related protein 1 on the blood-labyrinth barrier has opened up new avenues for therapeutics delivery in the inner ear.^5^
